# Automated Retinal Vessel Analysis Based on Fundus Photographs as a Predictor for Non-Ophthalmic Diseases—Evolution and Perspectives

**DOI:** 10.3390/jpm14010045

**Published:** 2023-12-29

**Authors:** Ciprian Danielescu, Marius Gabriel Dabija, Alin Horatiu Nedelcu, Vasile Valeriu Lupu, Ancuta Lupu, Ileana Ioniuc, Georgiana-Emmanuela Gîlcă-Blanariu, Vlad-Constantin Donica, Maria-Luciana Anton, Ovidiu Musat

**Affiliations:** 1 Department of Ophthalmology, University of Medicine and Pharmacy “Grigore T. Popa”, 700115 Iasi, Romania; ciprian.danielescu@umfiasi.ro; 2 Department of Surgery II, Discipline of Neurosurgery, University of Medicine and Pharmacy “Grigore T. Popa”, 700115 Iasi, Romania; marius.dabija@umfiasi.ro; 3 Department of Morpho-Functional Sciences I, University of Medicine and Pharmacy “Grigore T. Popa”, 700115 Iasi, Romania; alin.nedelcu@umfiasi.ro; 4 Department of Pediatrics, University of Medicine and Pharmacy “Grigore T. Popa”, 700115 Iasi, Romania; vasile.lupu@umfiasi.ro (V.V.L.); ileana.ioniuc@umfiasi.ro (I.I.); 5 Department of Gastroenterology, University of Medicine and Pharmacy “Grigore T. Popa”, 700115 Iasi, Romania; 6 Doctoral School, University of Medicine and Pharmacy “Grigore T. Popa”, 700115 Iasi, Romania; vlad_constantin.donica@d.umfiasi.ro (V.-C.D.); anton_maria-luciana@d.umfiasi.ro (M.-L.A.); 7 Department of Ophthalmology, University of Medicine and Pharmacy “Carol Davila”, 020021 Bucuresti, Romania; ovidiu.musat@umfcd.ro

**Keywords:** retinal vessel analysis, dynamic vessel analysis, cardiovascular risk, neurovascular coupling, artificial intelligence

## Abstract

The study of retinal vessels in relation to cardiovascular risk has a long history. The advent of a dedicated tool based on digital imaging, i.e., the retinal vessel analyzer, and also other software such as Integrative Vessel Analysis (IVAN), Singapore I Vessel Assessment (SIVA), and Vascular Assessment and Measurement Platform for Images of the Retina (VAMPIRE), has led to the accumulation of a formidable body of evidence regarding the prognostic value of retinal vessel analysis (RVA) for cardiovascular and cerebrovascular disease (including arterial hypertension in children). There is also the potential to monitor the response of retinal vessels to therapies such as physical activity or bariatric surgery. The dynamic vessel analyzer (DVA) remains a unique way of studying neurovascular coupling, helping to understand the pathogenesis of cerebrovascular and neurodegenerative conditions and also being complementary to techniques that measure macrovascular dysfunction. Beyond cardiovascular disease, retinal vessel analysis has shown associations with and prognostic value for neurological conditions, inflammation, kidney function, and respiratory disease. Artificial intelligence (AI) (represented by algorithms such as QUantitative Analysis of Retinal vessel Topology and siZe (QUARTZ), SIVA-DLS (SIVA—deep learning system), and many others) seems efficient in extracting information from fundus photographs, providing prognoses of various general conditions with unprecedented predictive value. The future challenges will be integrating RVA and other qualitative and quantitative risk factors in a unique, comprehensive prediction tool, certainly powered by AI, while building the much-needed acceptance for such an approach inside the medical community and reducing the “black box” effect, possibly by means of saliency maps.

## 1. Introduction

Examining the eye fundus in search of clues about the status of other organs and systems has a long history. Retinal-based quantification of cardiovascular disease risk began with the grading system of hypertensive retinopathy proposed by Keith and Wagener in 1939 [1]. Another traditional grading system that summarizes the ocular signs of hypertension was proposed by Scheie et al. in 1953. Ever since clinicians have been accustomed to incorporating the information provided by the fundus examination into their decision tree when managing arterial hypertension.

As cardiovascular disease remains a prevalent cause of global mortality, researchers are always seeking screening tools that are population-based and able to detect early, asymptomatic, subclinical signs in order to predict the risk of a certain individual. Incorporating retinal photographs into such research is a logical choice since it is a non-invasive technique, relatively easy to perform, and rich in information.

The first large population-based study that evaluated the relationship between retinal vasculature and cardiovascular disease by incorporating retinal photographs into the study protocol was the Atherosclerosis Risk in Communities study (ARIC). Retinal images were evaluated by certified graders who had been masked to participant characteristics [2].

The next step in the evolution of studying retinal vasculature was the advent of the retinal vessel analyzer, proposed by Seifertl and Vilser in 2002. At that time, it consisted of a fundus camera with a CCD measuring camera attached and an image-processing unit [3].

After this invention, measuring the retinal arteriolar and venular diameters on digitized images became part of many large epidemiological studies that have shaped our understanding of cardiovascular disease [4,5,6]. Gradually, the measurements became semi-automated (still needing the input of human graders) and then automated. Recently, advances in artificial intelligence research have allowed algorithms to draw information from complex images, paving the way for a new era where cardiovascular (and perhaps many other) conditions may be predicted with astonishing accuracy from fundus photography without the use of human labor.

In this study, we performed a review with respect to the use of retinal vessel analysis (based on fundus photographs) as a tool in the prevention, risk stratification, diagnosis, and management of non-ophthalmic diseases, starting with a search of MEDLINE (PubMed), Web of Science (Clarivate Analytics), and the Cochrane Library (Cochrane). Search strategies were constructed using database-specific subject headings and keywords. While the initial search was focused on studies published in the last 5 years (2019–2023), references in those studies provided us with a wide array of literature that helped us to comprehend the evolution of this diagnostic method better. We only selected articles regarding the analysis of fundus photographs. Other techniques, such as optic coherence tomography angiography, and adaptive optics imaging, have also been used to study retinal vasculature, but we have not included them in this review (there are a lot of studies published about these techniques, so perhaps they should be approached in another review).

## 2. Description of the Diagnostic Technique

The retinal vessel analyzer (RVA, commercialized by IMEDOS) was devised to measure the diameter of retinal vessels in relation to time and locations along the vessel. This software evaluates a digital fundus image using ring-shaped markers centered on the optic disc, making an automated preselection of all detected arterial and venous segments. It is based on the procedures described by Hubbard in the ARIC (Atherosclerosis Risk in Community) study and was further refined by Knudtson et al., using the six largest arterioles and venules to compute summarized retinal vascular measures [7,8]. It provides the central retinal arteriolar equivalent (CRAE), central retinal venular equivalent (CRVE), and the arteriolar-to-venular ratio (AVR) as the CRAE/CRVE ratio [9].

An interesting and useful concept, neurovascular coupling, refers to the regulation mechanism that links transient neural activity to the subsequent change in cerebral blood flow, while at the microscopic level, the neurovascular unit is composed of the vascular smooth muscle, the neuron, and the astrocyte glial cell [10]. It may be studied using a dynamic vessel analyzer (DVA, IMEDOS), today at the third generation, which measures the diameters of retinal vessels in real-time, first in a baseline state, then while the autoregulatory mechanisms are stimulated by flickering light for 20 s. The phases of stimulation and recovery are repeated two more times, and then signal averaging is performed. Vessel diameters of each vessel segment are recorded continuously over time. This requires the automatic correction of image irregularities; for example, caused by eye movements [11]. The three vascular parameters obtained are flicker-light-induced dilation of the artery (aFID), flicker-light-induced dilation of the vein (vFID), and flicker-light constriction of the artery (aFIC) [12]. The prognostic value of the DVA is presented in Section 5 and Section 6.

Since retinal photography is usually performed with the patient in a sitting position, but RVA would also be useful for prognostic purposes in patients who are confined to bed, a maneuverable RVA has been devised for examining supine patients (for instance, patients with acute subarachnoidal hemorrhage). However, because of the limited maneuverability of the fundoscope when a patient is in a supine position, there is also work in progress to develop a miniaturized, wearable device for RVA comparable to “smart glasses” [13].

There are several other software used for the analysis of retinal vasculature. Integrative vessel analysis (IVAN) is a semi-automated program for measuring retinal vessel diameter, which was created at the University of Wisconsin. It needs trained graders to perform measurements [14]. It was used in early epidemiologic studies such as the Wisconsin Epidemiologic Study of Diabetic Retinopathy and the Beaver Dam Eye Study, and later studies such as the Multi-Ethnic Study of Atherosclerosis, the Singapore Malay Eye Study, the Singapore Prospective Study Program, and the Age, Gene/Environment Susceptibility-Reykjavik Study [4,6,14,15].

Singapore I Vessel Assessment (SIVA, National University of Singapore) involves automated detection of the optic disc center and optic disc edge, as well as automated detection and identification of arterioles and venules. It also adds additional geometry parameters such as branching angles, bifurcation, fractal dimension, and tortuosity and may establish early indicators of microvascular damage [15,16]. For instance, arteriolar and venular tortuosity are correlated with mean arterial blood pressure [17,18]. This software accepts manual correction on vessel tracking. It was used in the Montrachet study.

VAMPIRE is the vessel assessment and measurement platform for images of the retina, a software application for semi-automatic quantification of retinal vessel properties, including vessel width, vessel branching coefficients, and tortuosity [19]. It was used in the Lothian Birth Cohort 1936 and in the Mild Stroke Study [20].

## 3. Normative Data and Normal Changes with Age

The Gutenberg Health Study provided sex- and age-specific normative data for retinal vasculature [21]. In all eyes, the means for CRAE, CRVE, and AVR were 178.37 ± 17.91 μm, 212.30 ± 17.45 μm, and 0.84 ± 0.07, respectively. Women had higher values of CRAE, central retinal venular equivalent, and AVR than men. The Gutenberg Study also provided reference ranges from the 2.5th to the 97.5th percentiles for CRAE, CRVE, and AVR for men and women aged ≤55 and over 55 years. Streese et al. published normative data from a population of healthy Swiss subjects (the COMPLETE Health Study). The means for CRAE, CRVE, and AVR were 181 ± 16 μm, 211 ± 16 μm, and 0.86 ± 0.06. The average decline per decade was 6 μm for CRAE and 4 μm for CRVE [22].

In children, development over 4 years of follow-up was associated with an average mean narrowing of retinal arterioles by 6 µm, and girls had wider CRAE and CRVE compared with boys [23]. These data were recently corroborated by another study on children aged 4 to 5 years, in which the average CRAE was 180.9 ± 14.2 µm (178 µm in boys and 182 µm in girls) and the average CRVE was 251 ± 19.7 µm (247 in boys and 254 in girls) [24]. Kochli et al. highlighted that, just as in adult populations, it is important to differentiate cardiovascular risks by microvascular phenotype in different populations and ethnicities early in life, as black South African children presented wider retinal venules when compared with their white South African and Swiss peers [25].

A normal DVA response features primary vasodilation upon initiation of flicker light excitation (FLE), which reaches a maximum after a characteristic latency and is followed, after termination of the stimulus, by vasoconstriction [13]. Normal expected retinal arterial responses to FLE are around 6.9 ± 2.8% [26,27]. Other authors have calculated more parameters such as baseline-diameter fluctuation (BDF), i.e., the difference between the maximum and minimum baseline vessel diameter; maximum diameter reaction time (MDRT), i.e., the time taken to reach the maximum vessel dilation in response to FLE; and dilation amplitude (DA), i.e., the difference between maximal dilation and constriction responses [28,29]. We are not aware of any DVA studies being performed on children.

For the first time, normative data for DVA parameters were derived from the COMPLETE Health Study: the overall means for aFID, vFID, and aCON (arteriolar constriction) were 3.65 ± 1.96%, 4.23 ± 1.84%, and −1.65 ± 1.5%; normative data for quantiles can also be found in that study [22]. The authors detected that vFID and arteriolar constriction (but not aFID) showed a negative association with age.

## 4. Concordance between Measurements Using Different Software

Retinal vasculature measurements using SIVA and VAMPIRE have shown poor to limited agreement [20]. Also, the agreement between VAMPIRE and IVAN was poor to moderate. However, that between IVAN and SIVA was considered good to excellent, with a mean difference of 16.4 µm for CRAE and 19.3 µm for CRVE. An algorithm was proposed for converting IVAN into SIVA-estimated measurements [30].

Wei et al. used a purpose-built microcontroller to generate a trigger pulse for acquiring a retinal image delayed by 300 ms relative to the top of the electrocardiographic (ECG)R-wave (obtaining what are usually known as ECG-gated images). Then, they assessed intra-grader repeatability between CRAE, CRVE, and AVR as measured using IVAN and SIVA. The values of CRAE and CRVE provided by SIVA were systematically larger than those provided by IVAN, while the AVR estimates were similar between the two programs. Repeatability was not improved with the use of ECG gating. The authors concluded that combining historical readings analyzed using IVAN with more recent readings by SIVA is possible only for AVR [17]. They also suggested that a possible way to pool CRAE and CRVE estimates obtained with IVAN and SIVA would be by expressing individual readings in units of standard deviation after stratification (another way would be to re-measure historical images using the newer software).

## 5. Automated Retinal Vessel Analysis and Cardiovascular Conditions

Retinal vessel analysis has been confirmed as a reliable indicator of cardiovascular risk by several large epidemiological studies. It was estimated that each 10 mmHg increase in mean blood pressure (BP) reduced the retinal arteriolar diameter by approximately 3 μm [11]. In the Atherosclerosis Risk in Communities Study (ARIC), a lower AVR was independently associated with a higher stroke risk after adjusting for other risk factors such as mean arterial pressure, total and HDL cholesterol, and glycemia [2]. In a recent analysis of ARIC participants who were free of cardiovascular disease at the baseline and were followed for a mean of 16 years, narrower CRAE and wider CRVE were associated with the incidence of heart failure, even after adjusting for other clinical risk factors. They were also associated with larger left ventricular size, higher prevalence of left ventricular hypertrophy, and worse measures of diastolic and systolic function [31], confirming the data regarding the prognostic value of RVA for potential heart failure from the Multi-Ethnic Study of Atherosclerosis (MESA) [32,33].

For participants in the Beaver Dam Eye Study who died of coronary heart disease or stroke, generalized arteriolar narrowing at baseline (defined as the lowest quintile of the AVR distribution) was associated with cardiovascular mortality in those who were considered younger (43 to 74 years) at baseline [34]. In the Rotterdam Study, lower AVR (calculated using Retinal Analysis semi-automated software) was associated with increased carotid intima-media thickness, higher carotid plaque score, and other cardiovascular risk factors such as lower HDL, higher body mass index, higher waist-to-hip ratio, and smoking [35]. The MESA confirmed the findings for the prognostic value of arteriolar narrowing, while retinal venular widening was also found to be independently associated with the development of hypertension [6]. In the Gutenberg Health Study, people with untreated or insufficiently treated hypertension were more likely to have retinal vessel equivalents outside the reference range [21].

Interestingly, in the Thessaloniki Eye Study, Dervenis et al. suggested that the negative correlation between BP and CRAE seems to be guided by the effect of diastolic BP, as higher systolic BP is independently associated with higher values of CRAE [36].

The importance of microvascular dysfunction as a major pathogenic factor for the development of high BP was further confirmed by a meta-analysis of six population-based cohort studies, which totaled 10,229 participants. Both narrower CRAE and wider CRVE were associated with an increased risk of hypertension. Each 20 µm narrower arterioles at baseline were associated with a 1.12 mmHg increase in BP over 5 years [37].

While associations of retinal vessel measurements with BP values and general cardiovascular risk appear to be very well documented, in a sample of the general population, smaller CRAE was associated with a higher calculated forward pulse (Pf), backward pulse (Pb), central pulse pressure, and pulse-wave velocity. In a stiff arterial tree, the backward wave returns during systole, augmenting central systolic blood pressure. The pulsatile stress on the wall of the large arteries leads to further degradation in the elastin fibers, leaving stiffer collagen fibers to bear the pulsatile load [38]. Also, in the SAFAR study, ambulatory aortic stiffness and office-measured carotid to femoral pulse-wave velocity were associated with CRAE [39].

Some authors have investigated the prognostic value of retinal vessel analysis in children. In a cross-sectional study of primary school children, high-normal BP or BP in the hypertensive range was associated with narrower CRAE (as well as higher pulse-wave velocity) when compared with normotensive peers [40]. In a longitudinal study of children (6 to 8 years old at baseline), narrower CRAE at baseline was associated with higher systolic BP after four years [23], while in the Young Finns Study, there was a strong negative association between childhood systolic BP and adult arteriolar diameter. Also, there was a positive correlation between childhood systolic BP and adult arteriolar tortuosity [41]. Thus, retinal vessel analysis may prove to be a valuable primary prevention screening tool in children.

Concerning dynamic analysis, in a recent observational study, patients with coronary artery disease or cardiovascular risk factors were analyzed with a DVA. Over a median period of 8.6 years, 36% of them developed major adverse cardiovascular events (MACE). After adjusting for traditional risk factors, patients within the lowest quintile of aFID had the highest risk of MACE, while vFID had no predictive value [42].

We must remember that retinal vessel analysis alone cannot offer information about the entirety of the vascular bed. Forearm venous occlusion plethysmography (FBF), one of the “gold standards” in the assessment of endothelial disfunction, was compared with DVA analysis in two groups of subjects (high-risk cardiovascular patients and healthy people), and the conclusion was that they were not equivalent as methods of testing microvascular function. The FBF response to acetylcholine was significantly blunted in the patient group, while the DVA did not detect any difference between groups [43].

Patel et al. investigated microvascular function (via DVA) in apparently healthy subjects with signs of macrovascular endothelial disfunction, measuring brachial artery reactivity via the flow-mediated dilation (FMD) technique. A reduction in FMD response was accompanied by reduced baseline-correctedaFID, reduced arteriolar maximum dilation, longer dilation reaction time, and lower dilation amplitude. It was concluded that early signs of vascular dysfunction are reflected simultaneously at both macro- and microvascular levels [26]. Reduced basal nitric oxide availability appears to be the common denominator of micro- and macrovascular endothelial dysfunction [44,45].

Retinal vessel analysis is also useful as a tool that highlights the effects of a therapeutical intervention. For instance, in sedentary subjects, after 12 weeks of high-intensity interval training, retinal arteriolar diameters increased, and retinal venular diameters decreased significantly, correlating with a reduction in plasmatic 3-nitrotyrosine, a marker of oxidative stress [46]. While obesity was associated with retinal arteriolar narrowing in the Atherosclerosis Risk in Community (even in the absence of arterial hypertension) study, CRAE became wider and AVR higher six weeks after bariatric surgery, and those changes were sustained (but not further improved) for 4 years [14,47]. Interestingly, indices of large artery stiffness, such as brachial–ankle pulse-wave velocity and cardio–ankle vascular index, did not change significantly when compared with pre-surgery values. In a systematic review of 21 articles, higher physical activity levels were associated with narrower CRVE in children and adults, while physical inactivity was associated with wider CRVE in both age groups. Aerobic exercise interventions in adults with or without cardiovascular risk factors induced wider CRAE and narrower CRVE [48]. It may be concluded that exercise has the potential to reverse or postpone the progression of small vessel disease in adults with cardiovascular risk [11].

## 6. Automated Retinal Vessel Analysis and Neurological Conditions

Stroke is a cerebrovascular condition that may be considered situated at the crossroads between vascular and neurological conditions. In a meta-analysis of six cohort studies that included more than 20,000 participants, wider CRVE predicted the advent of stroke (pooled hazard ratio = 1.15), while CRAE was not associated with stroke. The inclusion of the retinal venule caliber in prediction models led to the reassigning of 10% of people into different, mostly lower, categories [49].

A risk assessment model of ischemic stroke based on traditional risk factors combined with a classification of white matter lesions (on FLIR MRI) and with CRAE measurements provided by IVAN was proven to be superior to the traditional evaluation of risk factors and the single-index model [50].

While it is unanimously accepted that global arteriolar narrowing is associated with sclerosis of the arteriolar wall, a common feature of arterial hypertension, it remains significantly associated with brain ischemia even after adjustment for hypertension, diabetes, and other risk factors [51]. An association between retinal venular dilation and lacunar infarction, but not with other forms of cerebrovascular disease, seems more difficult to explain. It has been postulated that venular dilation reflects diffuse cerebral ischemia, which would mean that brain tissue is more prone to infarction [52].

A systematic review identified 11 reports that associated abnormalities in retinal vessel diameter and increased retinal vessel tortuosity with cerebral small vessel disease (CSVD), a progressive degenerative disorder of small-caliber cerebral vessels. However, the review underlined several shortcomings of existing evidence, such as a small sample size and an unstandardized approach to biomarker capturing and CSVD characterization [53]. Stronger retinal arteriolar vasoreactivity (as measured by DVA) was associated with more microcirculatory diffusivity (derived from intravoxel incoherent motion MRI or IVIM). The diffusivity corresponds to microvascular blood velocity and the architecture of the microvascular bed in normally appearing white matter and cortical gray matter [54]. There was no association between static arteriolar or venular measurements and IVIM. Another study (using SIVA) did not find an association between retinal vessel calibers and cerebral blood flow measured using arterial spin labeling MRI [55].

In a 10-year longitudinal study, increased retinal tortuosity as determined using SIVA software was associated with the incidence of all-cause dementia, while wider retinal calibers and higher venular tortuosity were associated with mixed or vascular dementia, but not Alzheimer’s disease [56]. Decreased arteriolar calibers were associated with decreased dementia risk in participants with diabetes, but not in those without diabetes. In a prospective study of 491 participants followed for 5 years, a deep learning model was used for the automated measurement of retinal vessel caliber from retinal photographs (the SIVA-DLS). Narrower arterial caliber was associated with incident dementia, while both narrower arteriolar caliber and wider retinal venular caliber were associated with an increased risk of cognitive decline [57].

Neovascular coupling in the retina is mediated by nitric oxide and eicosanoids, as is functional hyperemia in the brain [58]. Diminished flicker-induced arteriolar dilation may reflect direct damage to microcirculation or even neurodegeneration. Maximal arteriolar reaction was increased and dilation was delayed in patients with Alzheimer’s disease compared to healthy controls and to patients with mild cognitive impairment [59]. A DVA-based approach has been proposed as a means by which to evaluate the efficiency of therapeutic interventions targeting cellular mechanisms of microvascular aging [58].

An interesting study used a DVA to search for clues regarding the prediction of delayed cerebral ischemia (DCI), a common complication of aneurysmal subarachnoid hemorrhage. Analgo-sedated patients were examined in a supine position with the head and torso partially tilted, by means of a maneuverable RVA machine. Patients who developed DCI had a significantly shorter time to 30% maximum dilation (tMAD) on days 0–4 compared to patients without DCI, followed by a significantly higher tMAD on days 16–23 [13].

## 7. Automated Retinal Vessel Analysis in Rheumatological Conditions

In retinal photographs of rheumatoid arthritis patients, CRAE and AVR were inversely associated with carotid intima-media thickness (cIMT), a marker that is increased in subclinical atherosclerosis, and also with C-reactive protein (CRP). It is believed that inflammation is the biological link for this association [60]. Venular dilatation correlates with CRP levels, and it has been detected as a response to general surgery and also in patients with respiratory, urinary, or skin infections [61,62]. Therefore, the prognostic value of retinal vascular parameters is likely to be confounded if the patient suffers from acute inflammation.

In a pilot study of patients with rheumatoid arthritis (RA), primary Sjögren’s syndrome, and healthy controls, no differences were detected between groups regarding CRAE, CRVE, and AVR analyzed using Mona-Riva software [63]. However, other research groups have found an association between CRVE and the activity of rheumatologic diseases, even those with normal levels of C-reactive protein [64]. Also, pharmacological suppression of inflammation in RA was followed by a reduction in venular diameters [65]. Mild and moderate rheumatic disease activity is associated with an increase in retinal venular diameter (but no change in large artery stiffness measured via applanation tonometry), even in the absence of cardiovascular comorbidities. Regular physical activity significantly reduced venular diameters in those patients [66].

The AGES-Reykjavik, a population-based multi-disciplinary longitudinal cohort study of aging, found an association between retinal arterial narrowing and hand and knee osteoarthritis in males and females [67].

## 8. Automated Retinal Vessel Analysis in Other General Conditions

While variations in retinal vascular caliber have previously been reported in association with kidney disease, a meta-analysis of 11 studies that together included 44,803 participants found no significant associations between CRAE or CRVE and chronic kidney disease stages 3 to 5 in the general population [68].

Retinal arteriolar and venular diameters increase significantly after hemodialysis [69]. The ISAR (Risk Stratification in End-Stage Renal Disease) was performed on a cohort of multi-morbid patients on hemodialysis. aFID and vFID significantly predicted non-fatal cardiovascular events (hazard ratio 0.74) and also fatal cardiovascular events (hazard ratio 0.78) during a 44-month follow-up period [70]. In a more recent, longer follow-up study of hemodialysis patients (median period 73 months), retinal venular dilation exhibited a univariate hazard ratio for all-cause mortality of 0.69, which was even more pronounced for infection-related mortality (HR 0.53), while retinal arteriolar and venular diameters showed no predictive value [71].

In seniors, CRAE and estimated glomerular filtration rate (eGFR) decline in parallel with higher pulse pressure (systolic minus diastolic pressure), and CRAE < 150 µm identifies an early decline in eGFR [72]. It has been proposed that older patients with a pulse pressure of 70 mmHg should be referred for retinal imaging, and those with CRAE < 150 µm should be carefully monitored for the evolution of eGFR.

In patients with coronary artery disease and normal renal function, a decrease of 1% in flicker-induced retinal arteriolar dilatation was associated with an accelerated decline in eGFR of 0.07 mL/min/1.73 m^2^ (multi-variable analysis) [73].

The effect of BP on retinal vessels was also studied during pregnancy, taking advantage of the fact that RVA is a non-invasive, innocuous procedure. In the Growing Up in Singapore Towards Healthy Outcomes Study, quantitative retinal vascular parameters were assessed with the help of SIVA in 665 pregnant women. Every 10 mmHg increase in mean arterial blood pressure was associated with a 1.9 μm reduction in retinal arteriolar caliber, a 0.9° reduction in retinal arteriolar branching angle, and a 0.07 reduction in retinal arteriolar fractal dimension. Patients from a group classified as high-risk for preeclampsia were twice as likely to have generalized arteriolar narrowing, while retinal venular caliber and vascular tortuosity were not associated with BP measures [74]. In a cohort of Southeast Asian women, retinal arteriolar caliber and arteriolar and venular tortuosity were associated with a high risk of incident spontaneous abortion. More tortuous retinal vessels are assumed to be a feature of vascular inflammatory and oxidative stress [75]. Children from mothers who suffered from hypertensive pregnancy disorders had narrower arterioles in adulthood [76].

There have also been studies regarding the behavior of retinal vasculature in respiratory conditions. Using SIVA, McKay et al. demonstrated significant retinal arteriolar and venous dilation in chronic obstructive pulmonary disease (COPD) patients [77]. However, Vaes et al. found no association between retinal vessel parameters and lung function parameters in COPD patients. Also, an exercise-based pulmonary rehabilitation program did not affect retinal vessel diameters (but they were, of course, associated with systolic BP and systemic inflammation) [78].

When undergoing DVA analysis, individuals with untreated moderate-to-severe obstructive sleep apnea but without overt cardiovascular disease exhibited delayed reaction time in response to flicker, as well as decreased dilation amplitude, dilation slope, and post-flicker constriction, which can be early indicators for future vascular complications [79]. However, continuous positive airway pressure (CPAP) withdrawal did not affect retinal vascular responses to flickering light, suggesting that there are different effects on macrovascular and microvascular endothelial function [80].

In diabetic patients, higher arteriolar tortuosity was associated with the presence of early retinopathy, early kidney disfunction, a longer duration of diabetes, and reduced FLE vasodilatation [81].

## 9. Automated Retinal Vessel Analysis and Ocular Conditions

While this review is focused on the diagnostic capabilities of RVA for general disease, we must not forget that flicker-induced dilation of retinal vessels may be confounded by ophthalmic conditions, one of which is glaucoma. The general vessel response was shown to be reduced in primary open-angle glaucoma patients compared to normal controls and ocular hypertensive patients, and differences between the superior and inferior vessels appear to be correlated with asymmetrical altitudinal visual field defects [82,83]. Reduced vascular responsiveness is associated with increased peripapillary oxygenation exposure; therefore, ganglion cells and their axons in glaucomatous eyes with reduced vascular responsiveness are prone to be exposed to higher oxidative stress [84].

Both retinal arteriolar and venular diameters are reduced in retinitis pigmentosa, and the venular diameter is correlated with reduced multi-focal electroretinographic (ERG) amplitudes [85,86].

A possible confounding factor suggested by some authors is the fact that the use of pupil dilation with tropicamide and/or phenylephrine may affect retinal vessel measurements, especially endothelial NO-mediated retinal vasodilation [87]. Frost et al. found no change in the width of retinal vessels due to tropicamide, but they suggested that width parameters measured using standard approaches may be reduced due to image magnification resulting from cycloplegia. The same conclusion was supported by an optic coherence tomography angiography study [88]. Conversely, a small study by Wang et al. supports the hypothesis of a systematic effect of pupil dilation on retinal vessel caliber measurements [89].

## 10. Artificial Intelligence (AI) in Retinal Vessel Analysis

The term “artificial intelligence” encompasses both machine learning (which relies on manually labeled data) and deep learning (the ability to re-input the outputs of the initial learning in order to make regression decisions for final classification) [90,91,92].

A study that received significant attention was published by Poplin et al. in 2018. By training a convolutional neural network with more than 280,000 fundus photographs, the authors achieved the prediction of major cardiac events with an AUC of 0.70. Using the fundus photographs, their algorithm also managed to predict some other risk factors such as age (with a mean absolute error of 3.26 years), sex, and smoking status [93]. Interestingly, during the peer review of this study, the authors were asked to test the performance of the deep learning algorithm on a small dataset (239 patients) from a randomly selected Asian database, and its performance for all predicted cardiovascular risk factors was similar [94]. Since then, several other AI models for studying the association between retinal vasculature and general conditions have been developed.

QUantitative Analysis of Retinal vessel Topology and siZe (QUARTZ) is an automatic system that distinguishes between venules and arterioles, identifies vessel segments, outputs centerline coordinates, and measures vessel width and angular changes between vessel centerline coordinates while providing further measures of tortuosity [95,96]. It incorporates convolutional neural network technology and utilizes information from all vessels extracted within an image [97]. It was used in the European Prospective Investigation into Cancer (EPIC) study. When applied to data from the United Kingdom Biobank that included 68,550 participants aged 40 to 69 years who underwent nonmydriatic retinal imaging, blood pressure (BP), and arterial stiffness index assessment, it showed that greater arteriolar tortuosity was associated with higher systolic BP and higher pulse pressure, while narrower arterioles were associated with higher systolic BP, mean arterial pressure, and arterial stiffness index [97]. Also, associations between venular tortuosity and cardiometabolic risk factors differed according to diabetes status, while arteriolar diameter interacted significantly with BP, mean arterial pressure, and LDL cholesterol [98].

A deep learning model for the automated measurement of retinal vessel caliber from retinal photographs, known as SIVA-DLS (deep learning system), was trained on multi-ethnic, multi-country datasets comprising more than 70,000 images [99]. It used convolutional neural networks to estimate values of CRAE and CRVE in the region within 0.5–2 disc diameters away from the optic disc margin. The model performed comparably to or better than expert graders in identifying associations with cardiovascular disease factors.

Recently, a deep attention-based multiple-instance learning architecture was able to predict peripheral arterial disease from fundus photographs with an AUROC of 0.89. Analysis showed that the optic disc and the temporal arcades were weighted in order to obtain the results [100].

Another deep learning software package, the Retina-based Microvascular Health Assessment System (RMHAS), was compared with IVAN using a database of fundus photographs of Asian patients’ retinas. While the correlation was moderate for CRAE and AVR (ICC 0.62 and 0.42, respectively), it was good for CRVE (ICC 0.76) [101].

Werfel et al. recently developed an approach for untargeted machine learning on a DVA using noise reduction and extraction of independent components within DVA signals. Aiming to improve cardiovascular mortality prediction in a cohort of 214 hemodialysis patients, their technique resulted in the selection of a component highly correlated with maximal venous dilation following flicker stimulation (vMax), which was previously identified as a very strong predictor of mortality in that particular group of patients. It is expected that this type of machine-learning workflow should be useful for the analysis of DVA signals in other cardiovascular conditions [102].

An approach that seems to elicit significant interest is the prediction of biological age (BA). Deep learning algorithms have been able to predict this from fundus photographs [103,104,105]. The retinal age gap (the difference between biological age estimated from fundus photographs and chronological age) is significantly associated with the incidence of cardiovascular events and could stratify cardiovascular mortality risk. However, there is still high variability between populations and ethnic groups. Other authors have communicated associations between the retinal age gap and central obesity, metabolic syndrome, and inflammation [106,107].

A logical approach would be to enhance the prediction capabilities of AI by inputting more available risk factors that can be easily elicited from the patient. A simpler prognostic model based on retinal vasculometry (provided by QUARTZ), age, smoking status, and medical history (antihypertensive/cholesterol-lowering medication, diabetes, prevalent stroke/myocardial infarction) was used to predict the incidence of prediction of circulatory mortality, myocardial infarction, and stroke, performing equally or better than the Framingham risk score, a well-recognized method of risk-assessment [108].

Automatic retinal image analysis (ARIA) uses clues such as retinal vessel measurements, arteriole–venous nicking and arteriole occlusion, hemorrhages, tortuosity, bifurcation coefficients, asymmetry of branches, and bifurcation angles, which are studied using machine-learning-based methods [109]. This approach has been validated in different disease cohorts, including stroke, diabetes, and chronic kidney disease [110,111]. After ARIA learned to estimate age-related white-matter lesions (as detected on brain MRI) from fundus photographs, it predicted severe white-matter lesions with a sensitivity of 0.929 and a specificity of 0.984 [109].

Machine learning methods have also been successfully used to predict cerebral white matter hyperintensities (and even their location) based on fundus photographs [112,113]. As previously mentioned, SIVA-DLS was used in a prospective study on 491 participants followed for 5 years, confirming that narrower arterial caliber was associated with incident dementia, while narrower arteriolar caliber and wider retinal venular caliber were associated with an increased risk of cognitive decline [57].

It is obvious that deep learning software would analyze all elements present in a fundus photograph, not just the vascular diameters. For instance, we already know that arteriovenous nicking is associated with white-matter hyperintensities (WMHI) and lacunar infarction, focal arteriolar narrowing is associated with all subtypes of ischemic cerebrovascular disease, and retinal hemorrhages have shown a significant association with WHMI [52]. The association of microvascular abnormalities, grouped under the general term retinopathy, has shown a greater association with cerebrovascular diseases than any single retinal microvascular abnormality (that finding was compared with a dose–response effect). Artificial intelligence will surely consider all those retinal signs and likely find some other (discrete, subclinical) associations that we are not aware of today [114]. Of course, the ultimate goal is to integrate these measurements into a risk stratification tool that could assist physicians in identifying individuals who are more likely to develop a general condition in order to provide subsequent clinical management [115].

It is important to remember that AI models also have inherent biases. Similar to randomized trials, AI models may suffer from measurement bias (from confounders) and selection bias (from training and validation datasets). In order to diminish those biases, researchers must strive to select appropriate input datasets and output measures [116]. Also, similarly to randomized trials, information bias may be an issue when patients are required to input data (such as self-reported smoking or alcohol intake) [117]. Other sources of bias include data imbalance, distributional shifting, and domain generalization. The solution to the bias caused by unbalanced data would be to sample additional representative data. However, when training on retrospective data, this is not possible. Algorithmic solutions to this problem include using deep learning systems that require less training data using anomaly detectors or adversarial approaches [118].

Interestingly, it appears that AI dedicated to the interpretation of retinal images may suffer from specific biases; for instance, convolutional neural networks tend to be biased toward image textures and overfit to image details [100]. Also, assuming that skin pigmentation relates, on average, to the concentration of melanin within uveal melanocytes (and subsequently to retinal coloration) means that AI may become biased with respect to individuals of certain presumed races/ethnicities/origins [118] (Table S1).

## 11. Discussion

The interesting concept of oculomics relies on the characterization of macroscopic, microscopic, and molecular ophthalmic features associated with health and disease [1]. As we mentioned in the introduction, retinal vessels have been studied for a long time and there is robust evidence for the utility of retinal vessel analysis in order to quantify cardiovascular risk. While arteriolar measurements give strong information about arterial hypertension, risk of stroke, and coronary artery disease, venular measurements have been associated especially with obesity, hyperglycemia, and the risk of stroke [119].

Retinal microcirculation shares embryogenic, physiological, and morphological properties with vessels of the brain [11], including the presence of a blood–tissue barrier and auto-regulation of blood flow [54]. These considerations support the use of retinal vessel assessment as a marker for cerebral microvasculature, a non-invasive potential tool with which to screen and stratify individuals who may be susceptible to cerebral vascular disease but also cognitive decline and dementia.

Small-vessel disease includes reduced vasodilation, structural remodeling, and increased peripheral vascular resistance. It is an important mechanism in the pathophysiology of coronary artery disease, stroke, hypertensive retinopathy, and chronic kidney disease [11,120,121]. Narrow arterioles are the result of endothelial dysfunction and impairment of the L-arginine-nitric oxide (NO) pathway [23]. Diminished NO bioavailability, together with the release of endothelin-1 (ET-1), induces arteriolar constriction. Retinal venular constriction in response to ET-1 appears to be mediated by the activation of rho kinase signaling without the involvement of protein kinase C [122].

A recently published consensus study of the European Society of Cardiology Working Groups on Atherosclerosis and Vascular Biology, Aorta and Peripheral Vascular Diseases, Coronary Pathophysiology and Microcirculation, and Thrombosis stated that “Newer techniques to measure endothelial function that are relatively easy to perform, such as finger plethysmography and the retinal flicker test, have the potential for increased clinical use provided a consensus is achieved on the measurement protocol.” The consensus group recommended that further clinical studies should aim to establish reference values for these techniques [123]. Normative databases should be constructed for different populations and ethnicities and also for pediatric populations. Regarding the strategy, Hanssen et al. suggested that individuals with low and intermediate risk would benefit the most from add-on risk stratification through the use of vascular biomarkers since high-risk patients can already be reliably identified using classical risk assessment. RVA and DVA should be combined with a macrovascular biomarker of CV risk, such as the measurement of large-artery stiffness [11].

The use of non-invasive ophthalmic exploration in order to predict non-ophthalmic disease may benefit from the fact that the general population is very likely to attend an eye check regularly. For instance, half of the UK population attended an optometric practice in 2016. In contrast, a free check by primary care physicians aimed at the stratification of cardiovascular risk took 5 years to be accessed by 52% of the targeted population [124].

As several types of software have been developed for measuring retinal vessels, users are in the position of making an educated choice. RVA, IVAN, SIVA, and VAMPIRE are all semi-automated to various degrees. RVA and IVAN use ring-shaped markers (or a standardized ARIC grid) that have to be centered on the optic disc. For SIVA and VAMPIRE, the optic disc is identified automatically by the software and adjusted manually in case of incorrect location [30]. Measuring the vessels of the same eye with these software packages will yield different values, although conversion formulas have been proposed for the purpose of meta-analyses. However, the AVR is a parameter that does not change between different software [17]. Also, the strength of associations with systemic factors (e.g., blood pressure or cholesterol) and the measured vessel calibers are similar for different software [15,20]. SIVA and VAMPIRE offer more parameters, such as the tortuosity and fractal dimension of arterioles and venules, but these parameters have been studied less extensively. Also, DVA remains a “one of a kind” tool for exploring the dynamics of neurovascular coupling.

An important limitation of the predictive value of RVA is that, while there is a large consensus regarding the significant correlations between retinal vessel diameters and systemic CV risk factors in large-cohort studies, those correlations are often weak or moderate. This limitation may be foreseeably overcome by combining RVA with anamnestic risk factors and perhaps techniques that characterize large vessels in a single comprehensive prognostic algorithm.

Also, flicker-light-induced dilation has a high potential for clinical implementation in the primary and secondary prevention of chronic non-communicable diseases [11]. Of course, the currently available DVA device is still costly and requires skilled technicians as well as co-operative patients [11]. However, it has the potential to become a useful and largely accessible tool in the foreseeable future, as it has both extensive validation by the scientific community and potential for being useful in providing data for analysis by artificial intelligence.

New diagnostic techniques, such as retinal vessel wall analysis (assessment of the wall-to-lumen ratio by means of adaptive optics (AO) technology), need further validation from different study groups [125,126]. Other techniques that are widely used for ophthalmological diagnosis, such as optic coherence tomography angiography (OCTA) and, to a lesser extent, AO are also being evaluated in many published studies for the prognostic value of cardiovascular or other conditions, but we have not addressed that field of research in our review. A common criticism is that OCTA and AO are much more expensive devices when compared to a fundus camera, and this would be an obstacle to the widespread use of those techniques in screening settings.

As image acquisition devices (non-mydriatic fundus cameras, but also smartphone-based systems for fundus photography) become increasingly available, one might easily imagine a near future in which such an imaging device will be at the disposition of each physician (especially general practitioners) and the information provided would be integrated into a risk analysis generated by artificial intelligence. This would create a fast and effective way of personalizing the approach for each individual who is submitted for screening, emphasizing lifestyle and therapeutic interventions tailored for them. Also, AI might continue to improve by using increasing amounts of available data from those people, including the evolution of physical parameters in each individual. The “knowledge” accumulated by such an AI would be instantly updated and available to all clinicians and their patients.

A frequent issue highlighted by critics of the widespread use of AI is the fact that, in many cases, it is hard even for the creators of the software to understand the “logic” of the machine (in our setting, the question is, which features of the fundus photograph, perhaps unimportant even for the experienced grader, are used to achieve such high levels of prediction of cardiovascular events?). This is called “the black box issue.” Saliency maps are a method that highlights which region (or pixels) of the image contribute most to the algorithm’s decisions [127]. This is how it was understood that foveal morphology appears to be crucial in the machine’s ability to determine gender based only on fundus photographs.

The availability of databases of fundus photographs (combined with some clinical data regarding the respective patients) means that any new algorithm can be rapidly trained, tested, and then improved. The images used for training the AI should have enough ethnic variability, but too many images may decrease the efficacy of the training process. Also, data should be restricted to those criteria of greatest prognostic relevance; otherwise, the algorithms may become too complex [128].

There is still considerable delay regarding the external validation of the algorithms, a process that would mean extensive collaboration with clinicians (cardiologists, internists, ophthalmologists). That process would, in turn, build the necessary trust that the medical world should learn to put into those predictive tools. Last but not least, there is already evidence from cost-effectiveness analyses showing that AI, either standalone or used with humans, is more cost-effective than manual diabetic retinopathy (DR) screening and economic evaluation of AI for DR screening can be used as a model for the use of AI to predict other diseases [129].

Ophthalmology is fertile terrain for the development and future extensive clinical use of AI. In a recent comprehensive analysis of FDA-approved artificial intelligence and machine learning (AI/ML)-enabled medical devices, ophthalmology was classified as occupying the sixth place among medical subspecialties. There are nine FDA-approved AI devices in the ophthalmology subspecialty [130].

In conclusion, there is a large consensus regarding the significant (albeit moderate) correlations between retinal vessel diameters and systemic CV risk factors. There is also a body of promising research regarding the correlation of RVA with degenerative cerebral disorders, the evolution of renal function, normal and pathological pregnancy, or respiratory conditions such as sleep apnea. It appears that the most useful setting for RVA would be automated analysis (probably by means of AI) and its integration into a comprehensive risk assessment tool, together with other quantitative and qualitative risk factors. This approach would take advantage of the predictive value and large availability of retinal photography (perhaps even by means of a smartphone-based platform).

## Data Availability

Data sharing not applicable.

## Supplementary Materials

The following supporting information can be downloaded at https://www.mdpi.com/article/10.3390/jpm14010045/s1. Table S1. Studies of retinal vessels analysis as predictor for non-ophthalmic diseases.
