# Supplementary material for: Automated Retinal Vessel Analysis Based on Fundus Photographs as a Predictor for Non-Ophthalmic Diseases—Evolution and Perspectives
Source: J Pers Med. 2023 Dec 29;14(1):45. doi: 10.3390/jpm14010045 (PMC10817503; doi:10.3390/jpm14010045)
Supplement: Supplementary file 1 [file jpm-14-00045-s001.zip › jpm-2771327-supplementary.pdf]

**Table S1.** Studies of retinal vessels analysis as predictor for non-ophthalmic diseases.

| | Country | Retinal vessel analysis software | Subjects | Findings |
|---|---|---|---|---|
| Ponto 2017 [21] | Germany | RVA | 4309 | median CRAE and AVR were lower in participants with uncontrolled hypertension |
| Kotliar 2017 [59] | Germany | DVA | -5 patients with mild-to-moderate dementia due to Alzheimer's dementia (AD)<br>-24 patients with mild cognitive impairment due to AD (MCI)<br>-15 cognitively healthy controls (HC) | -maximal arterial reaction was increased and dilation was delayed in AD as compared to HC and MCI<br>- maximal venous reaction was increased in ADD as compared to HC and to MCI |
| Anyfanti 2017 [60] | Greece | Semiautomated software (Institute of Computer Science, Foundation for Research and Technology—Hellas) | -87 patients with rheumatoid arthritis<br>-46 controls | -among RA patients, CRAE and AVR were inversely associated with both carotid intima-media thickness (cIMT) and C-reactive protein (CRP), whereas CRVE positively correlated with CRP |
| Deiseroth 2018 [67] | Switzerland | RVA | 51 (patients with rheumatic diseases) and 35 controls | patients with rheumatic disease and mild-to-moderate disease activity show an impairment of the retinal microvasculature |
| McGrory 2018 [20] | United Kingdom | SIVA, VAMPIRE | 655 | SIVA and VAMPIRE measurements were associated most consistently with systemic variables relating to blood pressure |
| Tapp 2018 [76] | Finland | Semi-automated grading system | 1006 children and adolescents | Retinal arteriolar diameters were narrower and the arteriolar length-to-diameter ratio was higher among offspring of hypertensive pregnancies |
| Poplin 2018 [93] | | | | |

| | | | | |
|---|---|---|---|---|
| Dervenis 2019 [36] | Greece | IVAN | 1614 | negative correlation between BP and CRAE (due to the diastolic BP) |
| Wei 2019 [39] | Belgium | IVAN | 735 randomly recruited Flemish | smaller CRAE is associated with higher central pulse pressure, pulse wave velocity, calculated forward and backward pulse wave |
| Chandra 2019 [31] | United States | ARIC protocol for Retinal Vessel Analysis (Hubbard) | 10 629, mean follow-up 16 years (started in 1993-1995) | adjusted for age, gender, and race, CRVE widening and CRAE narrowing were associated with larger left ventricular size, higher prevalence of left ventricular hypertrophy, and worse measures of diastolic and systolic function |
| Köchli 2019 [40] | Germany | RVA | 1171 children | overweight and obese children had narrower CRAE, wider CRVE, and higher pulse wave velocity compared with normal-weight children |
| Babaoğlu 2019 [64] | Turkiye | IVAN | -47 rheumatoid arthritis (RA) patients<br>-32 systemic lupus erythematosus patients<br>-45 healthy controls | - CRVE was significantly increased in active RA patients<br>- RA-related inflammation may have systemic vascular effects even with normal levels of CRP |
| Sun 2019 [69] | China | IVAN | 202 patients on hemodialysis | CRVE correlated with baseline diastolic BP, HDL and LDL cholesterol LDLC and with changes in diastolic BP |
| Günthner 2019 [70] | Germany | DVA | 214 patients on hemodialysis | maximum venular dilation is a strong independent predictor for all-cause mortality in hemodialysis patients |
| Frost 2019 [87] | Australia | SIVA | 41 healthy subjects | tropicamide does not change the width of the retinal vessels |
| Owen 2019 [95] | United Kingdom | QUARTZ | 5497 | -increased venular tortuosity was associated with higher body mass index (BMI), hemoglobin A1c (HbA1c) level and prevalent type 2 diabetes<br>-wider venules were associated with older age, higher triglyceride levels, BMI , HbA1c level, and being a current smoker<br>-thinner venules were associated with high-density lipoprotein (HDL)<br>-arteriolar tortuosity increased with age, higher systolic BP, in females and in those with prevalent stroke ( |

| | | | | |
|---|---|---|---|---|
| | | | | -narrower arterioles were associated with age, higher systolic BP, total cholesterol level, and HDL |
| Tapp 2019 [97] | United Kingdom | QUARTZ | 68 550 | -greater arteriolar tortuosity was associated with higher systolic BP, higher mean arterial pressure and higher pulse pressure (PP)<br>-narrower arterioles were associated with higher systolic BP, higher mean arterial pressure, PP and arterial stiffness index |
| Schirutschke 2020 [44] | Germany | DVA | 23 high risk cardiovascular patients and 17 healthy persons | forearm blood flow and DVA are not equivalent for the testing of microvascular function |
| Lona 2020 [23] | Switzerland | RVA | 262 children (followed for 4 years) | narrower CRAE at baseline predicted higher systolic BP after 4 years |
| Shokr 2020 | United Kingdom | DVA | 201 normal /early hypertensives | microvascular alterations can be identifiable at BP values still considered within normal values and go in parallel with the changes observed in the level of oxidative stress. |
| Madhloum 2020 [24] | Belgium | MONA | 288 children (4 to 6-year old) | reference values for CRAE and CRVE |
| Nadal 2020 [55] | France | SIVA | 26 | cerebral blood flow is positively associated with venular fractal dimension |
| McKay 2020 [77] | United Kingdom | SIVA | 24 chronic obstructive pulmonary disease patients (COPD)<br>22 controls | COPD participants had wider mean arteriolar and venular diameters |
| Vaes 2020 [78] | Netherlands | MONA | 196 patients from an exercise-based pulmonary rehabilitation program | no associations between retinal vessel diameters and lung function parameters or functional outcomes. |
| Zhao 2021 [51] | China | IVAN | 296 (128 cases of ischemic stroke and 168 controls) | the risk assessment model of ischemic stroke combined with Fazekas grade of white matter lesions and CRAE is superior to the traditional risk model and the single-index model |
| Albanna 2021 [13] | Germany | DVA | 70 (with subarachnoid hemorrhage) | aneurysmal subarachnoid hemorrhage results in sustained impairment of neurovascular coupling in the retina |
| Streese 2021 [22] | Switzerland | RVA, DVA | 277 healthy individuals | -establishing normative data |

| | | | | |
|---|---|---|---|---|
| | | | | - higher blood pressure was associated with narrower CRAE but higher arteriolar flicker induced dilation |
| Theuerle 2021 [42] | Australia | DVA | 252 | patients within the lowest quintile of flicker light-induced retinal arteriolar dilation had the highest risk of major adverse cardiovascular events |
| Fitt 2021 [62] | Australia | IVAN | 41 | in severe infections CRVE decreased as the infections resolved and CRP levels fell after antibiotic treatment |
| Kuerten 2021 [83] | Germany | DVA | 12 glaucoma patients | vessel reaction was higher in the hemisphere corresponding to the more advanced visual field defect. |
| Cheung 2021 [99] | | SIVA-DLS | multiethnic multicountry dataset of more than 70,000 retinal photographs | the models performed comparably to or better than expert graders in associations between measurements of retinal-vessel caliber and CVD risk factors, including blood pressure, body-mass index, total cholesterol and glycated-haemoglobin levels |
| Mautuit 2022 [121] | France | IVAN, VAMPIRE, Adaptive Optics | 20 | -the correlation VAMPIRE-AO (Adaptive Optics) is poor<br>-the correlation IVAN-AO is stronger (but seems to underestimate arterial diameters) |
| Türksever 2022 [85] | Switzerland | RVA | 34 patients with POAG and 17 controls | reduced venular responsiveness is associated with increased peripapillary oxygenation exposure (prone to higher oxidative stress) |
| Patel 2022 [26] | United Kingdom | DVA | 100 | individuals exhibiting reduced flow-mediated dilation responses (a macrovascular parameter) showed a reduced baseline-corrected microvascular arterial dilation response to flickering light |
| Köchli 2022 [25] | South Africa, Switzerland | RVA | 929 black and white South African (SA) and 1171 Swiss children (aged 5-9 years) | higher body mass index and BP associated with narrower CRAE in all children, except for BMI in black SA children |
| Grogan 2022 [62] | Australia | IVAN | 68 patients that underwent surgery | CRAE and CRVE increased postoperatively |
| Mautuit 2022 [30] | France | SIVA, IVAN, VAMPIRE | 133 | -the agreement between VAMPIRE and IVAN was poor to moderate<br>- the agreement between VAMPIRE and SIVA was poor to moderate |

| | | | | |
|---|---|---|---|---|
| | | | | -the agreement between IVAN and SIVA was good to excellent |
| van Dinther 2022 [55] | Netherlands | DVA | 70 | variations in retinal arteriolar function are linked to cerebral microvascular properties (assessed by the intravoxel incoherent motion in brain MRI) |
| Cheung 2022 [57] | Singapore | SIVA-DLS | 491 | in multivariable models, narrower retinal arteriolar caliber and wider retinal venular caliber were associated with increased risk of cognitive decline |
| Günthner 2022 [71] | Germany | DVA | 214 | retinal venular dilation (but not arteriolar and venular diameters) is a biomarker for risk prediction in patients with end-stage renal disease |
| Theuerle 2022 [73] | Australia | | 253 subjects with cardiovascular disease risk factors | in patients with normal renal function, subjects with the lowest flicker-induced arterial dilation responses exhibited the greatest annual decline in glomerular filtration rate |
| Li 2022 [75] | Singapore | SIVA | 1032 women | increased risk of spontaneous abortion among Asian women with more tortuous retinal vessels (assessed during the preconception phase) |
| Seshadri 2022 [79] | United Kingdom | DVA | -14 subjects with moderate to severe obstructive sleep apnoea (OSA)<br>-14 controls | delayed reaction time in response to flicker, decreased dilation amplitude, dilation slope and post-flicker constriction in OSA patients |
| Turnbull 2022 [80] | United Kingdom | DVA | -19 patients randomized to continuous positive airway pressure (CPAP)<br>-18 patients randomized to sham CPAP | CPAP withdrawal and a return of OSA had no significant effect on retinal microvascular responses |
| Tapp 2022 [98] | United Kingdom | QUARTZ | 50233 | for arteriolar diameter, significant interactions according to diabetes status were evident for systolic BP, diastolic BP, mean arterial pressure (MAP) and LDL-cholesterol |
| Mueller 2022 [100] | Germany | Multiple Instance Learning (MIL) architecture | 135 | ROC AUC score of 0.890 for predicting peripheral arterial disease |

| | | | | |
|---|---|---|---|---|
| Rudnicka 2022 [108] | United Kingdom | QUARTZ | 88 052 UK Biobank (UKB) and 7411 (EPIC)-Norfolk participants | retinal vasculometry model performed equally or better than Framingham risk scores (FRS) for incident stroke and incident myocardial infarction |
| Karimzad 2023 [29] | United Kingdom | VesselMap | 29 (subject to bariatric surgery) | CRAE and CRVE increased after bariatric surgery comparing to the baseline values |
| Reboucas 2023 [56] | France | SIVA | 584 | -increased retinal arteriolar tortuosity was associated with all-cause dementia<br>- wider retinal calibers and a higher venular tortuosity were associated with mixed/vascular dementia, but not Alzheimer's disease |
| He 2023 [101] | China | Retina-based Microvascular Health Assessment System (RMHAS) compared with IVAN | 4205 | ICCs were moderate for CRAE and AVR and excellent for CRVE |
